# TRIM5α is a SUMO substrate

**DOI:** 10.1186/s12977-015-0155-7

**Published:** 2015-03-24

**Authors:** Jacques Dutrieux, Débora M Portilho, Nathalie J Arhel, Uriel Hazan, Sébastien Nisole

**Affiliations:** INSERM UMR-S 1124, Université Paris Descartes, 45 rue des Saints-Pères, 75006 Paris, France; INSERM U941, University Institute of Hematology, Saint-Louis Hospital, 75010 Paris, France; LBPA, CNRS UMR 8113, Ecole Normale Supérieure de Cachan, 94235 Cachan, France

**Keywords:** TRIM5α, SUMO, Restriction factor, HIV-1

## Abstract

**Background:**

The TRIM5α restriction factor interferes with retroviral infections by inhibiting an early step of viral replication. TRIM5α activity was recently proposed to be regulated by the SUMO machinery and one SUMO consensus conjugation site as well as three putative SUMO interacting motifs (SIMs) were identified within TRIM5α sequence. Whereas mutation of the SIM sequences was found to abolish TRIM5α antiviral activity, mutation of the consensus SUMO conjugation site did not affect its restriction capacity, although this putative site has never been shown to be actually a SUMO substrate.

**Findings:**

Here we further demonstrate that TRIM5α relies on the SUMO machinery to promote restriction, since SUMO1 overexpression enhances TRIM5α-mediated retroviral inhibition whereas knockdown of SUMO1 or E2 SUMO conjugating enzyme Ubc9 prevents restriction. Furthermore, we show for the first time that TRIM5α is SUMOylated both *in vitro* and *in cellulo* and that Lysine 10 is the main SUMOylation site. Mutation of the consensus SUMO conjugation motif in position 10 abrogated SUMOylation at this position, but did not disrupt TRIM5α antiviral activity.

**Conclusions:**

Altogether, our results confirm that the SUMO machinery is involved in TRIM5α-mediated retroviral restriction, and demonstrate that TRIM5α is a SUMO 1 and SUMO 2 substrate. The inability to abrogate TRIM5α antiviral activity by mutating its main SUMO conjugation motif supports the notion that non-covalent interaction with SUMO or SUMOylated proteins rather than TRIM5α direct SUMOylation is required.

## Findings

TRIM5α is a restriction factor that interferes with an early step of retroviral replication in a species-dependent manner. Rhesus macaque TRIM5α (rhTRIM5α) is a potent inhibitor of HIV-1 infection, whereas its human counterpart (hTRIM5α) only weakly restricts HIV-1 [1,2]. TRIM5α is a cytoplasmic protein belonging to the TRIpartite Motif protein family (TRIM), a family of proteins characterized by their conserved structure composed, from N to C-terminus, by a RING domain, one or two B-Boxes and a Coiled-coil domain [3]. TRIM proteins mainly differ from each other by the nature of their C-terminal domain(s), which is a B30.2/SPRY domain in the case of TRIM5α. This domain allows TRIM5α to bind the viral capsid lattice of incoming particles and is therefore responsible for its species-specific activities.

Most TRIM proteins, including TRIM5α, self-associate into corpuscules, either cytoplasmic or nuclear [4]. One of the best-characterized members of this protein family is TRIM19, better known as promyelocytic leukemia protein (PML). TRIM19/PML is expressed in the nucleus, both in the nucleoplasm and in discrete subnuclear compartments known as PML nuclear bodies (reviewed in [5]). Modification of TRIM19/PML by small ubiquitin-related modifier (SUMO) has been demonstrated to modulate its stability, sub-nuclear localization and functions [5,6].

SUMOs are small ubiquitin-like proteins that are posttranslationally conjugated to other proteins, thereby regulating a wide range of biological processes, including transcription, DNA repair, chromosome dynamics and subcellular localization of target proteins (reviewed in [7,8]). The SUMO family consists of four members, termed SUMO1 to 4. SUMO is conjugated to protein substrates *via* an ATP-dependent enzymatic pathway involving 4 enzymes, a SUMO protease, an E1-activating enzyme, an E2-conjugating enzyme (Ubc9) and an E3 ligase, *via* a mechanism essentially analogous to that of ubiquitin [7,8]. Besides SUMOylation *per se*, proteins can also interact non-covalently with SUMO or SUMO-conjugated proteins *via* a SUMO-interacting motif (SIM) [7]. Three SIMs have been identified within TRIM5α B30.2 domain. Interestingly, mutation of two of these SIMs was shown to abolish TRIM5α-mediated restriction [9,10]. It is still unknown, however, whether TRIM5α is covalently modified by SUMO and whether SUMO conjugation could affect its activity.

**Figure 1:**
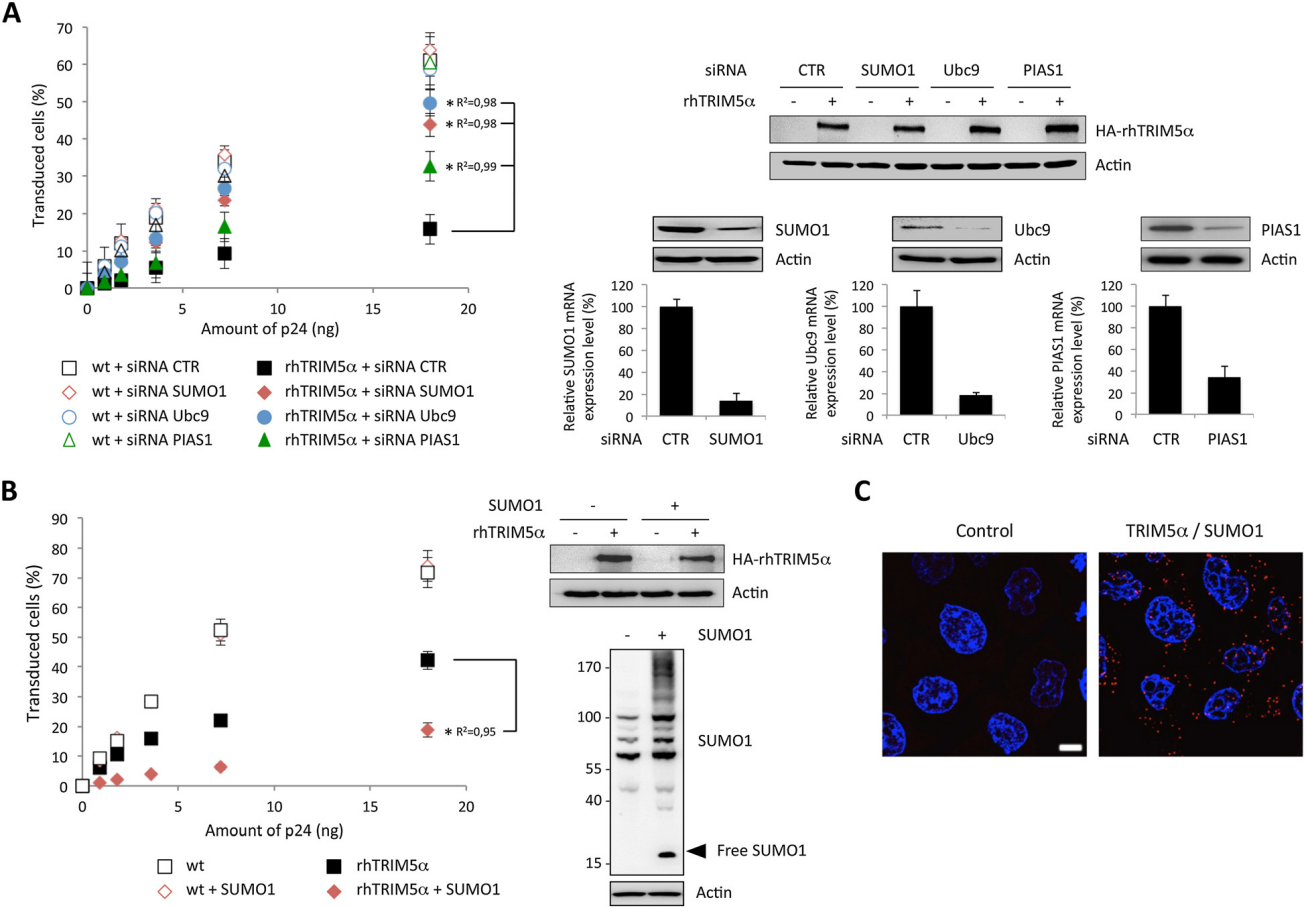
**SUMOylation regulates rhTRIM5α-mediated HIV-1 restriction. A**. HeLa cells were transfected or not with rhTRIM5α and with control siRNA or siRNA targeting SUMO1, Ubc9 or PIAS1, 48 h before being challenged by increasing doses of HIV-1/GFP. The proportion of GFP-positive cells was measured by flow cytometry 48 h post-transduction. The graph shows means of duplicate values +/- SD, representative of three independent experiments. *p < 0.001 using the Huber/White/sandwich variance-covariance robust estimator (linear regression coefficients R^2^ are indicated). The expression levels of HA-rhTRIM5α were evaluated by western-blot (top right) using anti-HA antibodies (3 F10, Roche). The efficiency of RNA silencing was estimated by quantitative RT-PCR analysis and western-blot using anti-SUMO1 (Santa-Cruz Biotechnology sc-9060), anti-Ubc9 (Abgent, AM1261a) and anti-PIAS1 (Abcam, ab32219) antibodies (bottom right). **B**. Wild-type or rhTRIM5α-expressing HeLa cells were transfected with His-SUMO1 before transduction with increasing doses of HIV-1/GFP. The proportion of GFP-positive cells was measured by flow cytometry 48 h post-transduction. The graph shows means of duplicate values +/- SD, representative of three independent experiments. *p < 0.001 using the Huber/White/sandwich variance-covariance robust estimator. The expression levels of HA-rhTRIM5α were evaluated by western-blot (top right). Cell extracts from HeLa untransfected or transfected with SUMO1 were also analyzed by western-blot for SUMO1 expression (bottom right). **C**. hTRIM5α interacts with SUMO1 in the cytoplasm and nuclei of HeLa cells. Paraformaldehyde-fixed cells were incubated with primary rabbit anti-TRIM5α [13] and mouse anti-SUMO1 (clone 21C7, DSHB) antibodies for 1 h at 37°C. After washing slides were incubated with Duolink PLA probes (Olink Biosciences) for 1 h at 37°C. Ligation of the connector oligonucleotides, rolling-circle amplification and detection of the amplified DNA products were done with Duolink Detection Reagents Red according to the manufacturer’s instructions. Nuclei were labelled with Hoechst. Images were acquired using an LSM510 inverted microscope with a 63× objective. Scale bar indicates 5 μm.

We first evaluated the implication of SUMO machinery in TRIM5α-mediated antiviral activity. For this purpose, HeLa cells were transfected or not with small amounts of rhTRIM5α-expressing plasmid, in order to obtain a moderate block of viral infection that can be modulated. We tested the effect of siRNA-mediated knockdown of endogenous SUMO1, Ubc9 (the E2 SUMO conjugating enzyme) or PIAS1 (an E3 SUMO ligase) expression on rhTRIM5α anti-HIV activity. As shown in Figure 1A, transfection of HeLa cells with control siRNA or with siRNA targeting SUMO1, Ubc9 or PIAS1 did not have any effect on HIV-1 infection. In contrast, we observed an increase in HIV-1 infection with siRNA targeting SUMO1 or Ubc9 in cells expressing rhTRIM5α (Figure 1A). Silencing of PIAS1 partially reversed rhTRIM5α-mediated HIV-1 restriction, suggesting that this E3-SUMO ligase is also likely to be implicated in TRIM5α restriction.

In order to confirm these results, we tested whether SUMO1 was capable of modulating rhTRIM5α-mediated HIV-1 restriction by transfecting SUMO1 in HeLa cells expressing or not rhTRIM5α. The expression level of rhTRIMα was reduced compared to panel 1A, in order to limit the amplitude of HIV-1 restriction. As shown in Figure 1B, overexpression of SUMO1 did not have any effect on HIV-1 infection of HeLa cells. In contrast, in rhTRIMα expressing cells, expression of SUMO1 led to a further decrease in HIV-1 infection. This observation clearly demonstrates that SUMO1 overexpression enhances the anti-HIV activity of rhTRIM5α. Altogether, these observations indicate that the SUMO machinery regulates rhTRIM5α-mediated restriction of HIV-1. This could indicate either that TRIM5α SUMOylation is required for HIV-1 restriction or that TRIM5α needs other proteins to be SUMOylated to display its antiviral activity.

To confirm interaction of TRIM5α with SUMO or SUMOylated proteins, we performed Duolink *in situ* proximity ligation assay (PLA). Cells were probed with anti-TRIM5α and anti-SUMO1 primary antibodies, followed by species-specific PLA probes. Our results show close proximity of TRIM5α with SUMO1, with an average of 38.6 spots per cell +/- 8.9 (SD), compared with 0.41 spots per cell +/- 0.39 for the PLA probes alone control. These results confirm that endogenous TRIM5α is conjugated by SUMO or interacts with SUMOylated proteins. Similar results were obtained following infection with HIV-1 (data not shown).

**Figure 2:**
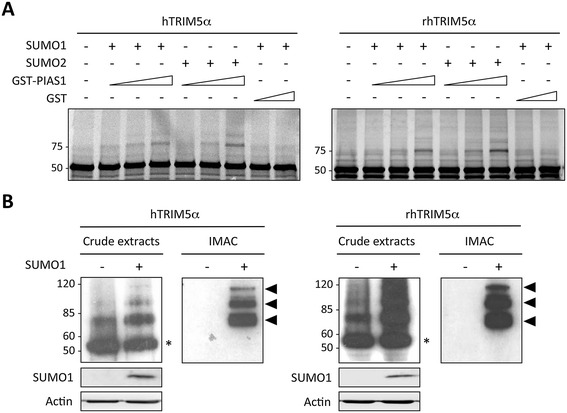
**Human and rhesus macaque TRIM5α are SUMO substrates. A**. *In vitro* translated human or rhesus macaque TRIM5α proteins were incubated with purified recombinant E1 (SAE1 and SAE2 subunits, 370 nM), E2 (Ubc9, 630 nM) enzymes, recombinant SUMO1 or SUMO2 (7 μM), and increasing doses of GST-PIAS1 (0, 0.3 or 1.5 μM) or GST alone (0.3 or 1.5 μM) as control. Reaction products were analyzed by 10% SDS-PAGE. **B**. HeLa cells expressing HA-tagged hTRIM5α or rhTRIM5α were transfected with Ubc9 and 6xHis-SUMO1, when indicated (+). Cell lysates were directly analyzed by SDS-PAGE and immunoblotted with anti-HA antibodies. Alternatively, cell extracts were subjected to an immobilized-metal affinity chromatography (IMAC) on nickel-agarose beads under strongly denaturing conditions and 6xHis-SUMO conjugated proteins were analyzed by SDS-PAGE and immunoblotted with anti-HA antibodies. The position of unmodified TRIM5α is indicated by an asterisk whereas black arrows indicate the position of SUMOylated forms.

In order to determine whether TRIM5α itself can be a substrate for SUMO modification, we employed an *in vitro* SUMOylation assay using purified recombinant E1 (SAE1 and SAE2 subunits) and E2 (Ubc9) enzymes, recombinant SUMO1 or SUMO2, and increasing doses of GST-tagged PIAS1 E3-ligase or GST alone, as control. As a substrate, we used *in vitro* translated ^35^S-labelled human or rhesus macaque TRIM5α. As shown in Figure 2A, in the absence of SUMO, a single band of 55 kDa corresponding to the unmodified TRIM5α can be observed. When SUMO1 or SUMO2 was added to the reaction, an additional band of approximately 75 kDa can be detected and corresponds to the expected size for TRIM5α conjugated to one SUMO1 moiety, thus demonstrating that both human and rhesus macaque TRIM5α proteins can be SUMOylated *in vitro*. Interestingly, we observed an enhancement of SUMO1 and SUMO2 conjugation to both human and rhesus macaque TRIM5α when the reaction was performed with increasing concentrations of GST-PIAS1.

Next, we sought to verify whether TRIM5α could also be SUMOylated *in cellulo*. Since no high molecular weight form of TRIM5α could be detected in wt HeLa cells (not shown), we co-transfected human or rhesus macaque HA-tagged TRIM5α with Ubc9 in order to boost the SUMOylation machinery. Two days post-transfection, cell extracts were analyzed by western-blot, revealing two bands, a major band at 55 kDa, which corresponds to unmodified TRIM5α, and a faint band at approximately 75 kDa, which corresponds to the expected size for TRIM5α conjugated to one SUMO moiety (Figure 2B). Interestingly, transfection of 6xHis-SUMO1 globally enhanced the proportion of high molecular weight bands, clearly revealing the existence of 3 species at approximately 75, 95 and 115 kDa (Figure 2B). These three slow-migrating bands of decreasing intensity may correspond to TRIM5α conjugated to one, two or three SUMO1 moieties, respectively. Alternatively, the higher molecular weight bands may represent mono-SUMOylated TRIM5α with other post-translational modifications, such as ubiquination, ISGylation, or neddylation. In order to verify whether these bands correspond to SUMOylated forms of TRIM5α, cell lysates were prepared under strongly denaturing conditions and proteins conjugated to 6xHis-SUMO1 were purified by immobilized-metal affinity chromatography (IMAC). Bound proteins were separated by SDS-PAGE and the presence of TRIM5α-HA was detected by western-blot (Figure 2B). As expected, three distinct bands can be detected at 75, 95 and 115 kDa, thus confirming the existence of 3 distinct SUMOylated forms of TRIM5α in SUMO1 overexpressing cells.

**Figure 3:**
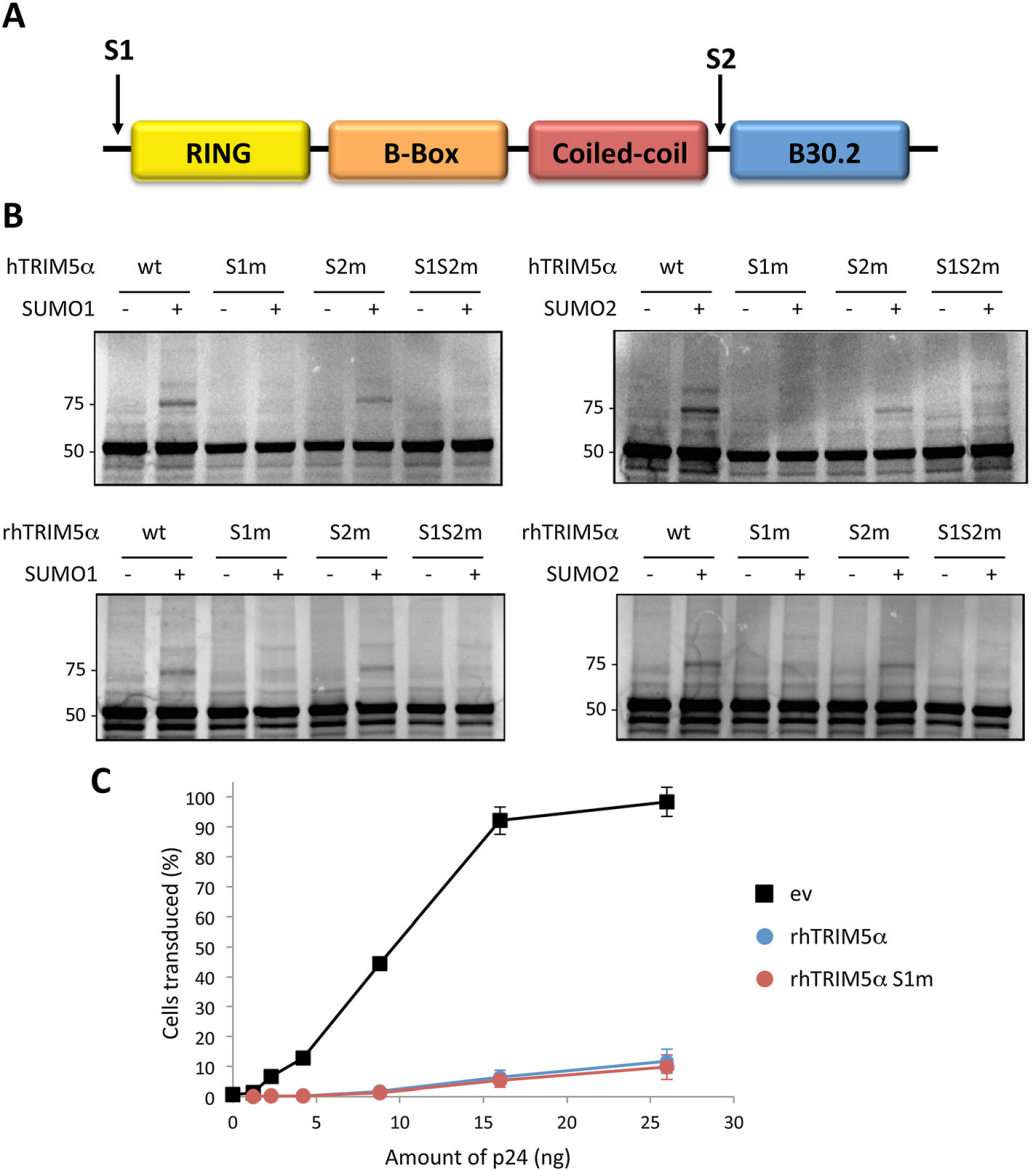
**TRIM5α** **is SUMO-modified on lysine 10. A**. Schematic representation of human and rhesus macaque TRIM5α proteins, indicating the predicted SUMOylated sites, S1 and S2. **B**. Wild-type hTRIM5α, rhTRIM5α, or the mutants S1m, S2m and S1S2m were *in vitro* translated, ^35^S-labelled and used as a substrate for *in vitro* SUMO-conjugation assay as described in Figure 2. Reaction products were analyzed by SDS-PAGE. **C**. HeLa cells were transfected with empty vector (ev), wt or S1m rhTRIM5α and transduced two days later with increasing concentrations of HIV-1/GFP. After 48 hours, the proportion of GFP-positive cells was measured by flow cytometry. Graphs shows means of triplicate values +/- SD, representative of three independent experiments.

For most target proteins, SUMOylation occurs on a conserved consensus motif ΨKXE (where Ψ is a hydrophobic amino acid) [7,8]. TRIM5α sequences contain two predicted SUMOylation sites, VK_10_EE (S1) and LK_263_KP (S2) in hTRIM5α, and VK_10_EE (S1) and LK_265_KP (S2) in rhTRIM5α. The first potential SUMO-conjugation site, referred to as S1, is located at the N-terminal extremity of the protein, before the RING domain, whereas the second putative site, S2, is located between the coiled-coil and the B30.2 domain (Figure 3A).

To determine whether either of these lysines are acceptor sites for SUMO1 conjugation, two single K to R point mutants were generated in human and rhesus macaque TRIM5α at K10 (S1m) and K263 or K265 (S2m), as well as double mutants (S1S2m). These constructs were tested in the *in vitro* SUMOylation assay. As shown in Figure 3B, the 75 kDa band can still be detected with S2m but not with S1m or with the double mutant, thus demonstrating that K10 is the major SUMOylation site within TRIM5α from both species. Identical results were obtained when the SUMOylation reactions were performed in the presence of SUMO2 instead of SUMO1 (Figure 3B).

Finally, we looked at the phenotype of S1 mutants by testing their capacity to restrict retroviral infections. Despite dramatic SUMOylation defects *in vitro*, rhTRIM5α S1m showed the same anti-HIV-1 activity than the wild-type protein, thus demonstrating that TRIM5α SUMOylation is not required for retroviral restriction, at least in HeLa cells (Figure 3C).

Although TRIM5α was suspected to be SUMOylated and that K10 was identified as a SUMOylation consensus site [9-11], TRIM5α SUMOylation has never been observed, neither *in cellulo* nor *in vitro*. This report is the first to demonstrate that TRIM5α is a SUMO substrate and to experimentally confirm that K10 is the main SUMOylation site. Previous groups have attempted to detect TRIM5α SUMOylation but without success [9-11]. Indeed the demonstration of TRIM5α SUMOylation by *in vitro* nickel pulldown assay required optimization of SUMO and Ubc9 expression and of binding conditions, which together may point to the fact that SUMOylation is transitory and affects only a small proportion of a given protein. Since rhTRIM5α K10R mutant is still able to restrict HIV-1, our results support the fact that the capacity of TRIM5α to interact with SUMOylated proteins, *via* its SIMs, rather than its direct SUMOylation, is required for its antiretroviral activity [9,10]. However, what is true for transfected TRIM5α is not necessarily applicable to the endogenous protein, particularly since endogenous TRIM5α is present in very small quantities. Indeed, it is possible that TRIM5α SUMOylation is important in certain cell types, to control TRIM5α activity and/or sub-cellular localization. We previously reported that dendritic cells from rhesus macaques are unable to restrict HIV-1, even if they express TRIM5α [12]. We are now conducting experiments to investigate whether this lack of restriction could be due to TRIM5α SUMOylation status.
